# Moral Attitudes Predict Cheating and Gamesmanship Behaviors Among Competitive Tennis Players

**DOI:** 10.3389/fpsyg.2017.00571

**Published:** 2017-04-12

**Authors:** Fabio Lucidi, Arnaldo Zelli, Luca Mallia, Giampaolo Nicolais, Lambros Lazuras, Martin S. Hagger

**Affiliations:** ^1^Department of Social and Developmental Psychology, Sapienza University of RomeRome, Italy; ^2^Department of Movement, Human and Health Sciences, Foro Italico University of RomeRome, Italy; ^3^Department of Psychology, Sociology and Politics, Sheffield Hallam UniversitySheffield, UK; ^4^School of Psychology and Speech Pathology, Curtin University, PerthWA, Australia; ^5^Faculty of Sport and Health Sciences, University of JyväskyläJyväskylä, Finland; ^6^Department of Physical Education, Hong Kong Baptist UniversityHong Kong, Hong Kong

**Keywords:** cheating, gamesmanship, moral attitudes, sport values, task and ego orientation, tennis

## Abstract

**Background:** The present study tested Lee et al.’s (2008) model of moral attitudes and cheating behavior in sports in an Italian sample of young tennis players and extended it to predict behavior in actual match play. In the first phase of the study we proposed that moral, competence and status values would predict prosocial and antisocial moral attitudes directly, and indirectly through athletes’ goal orientations. In the second phase, we hypothesized that moral attitudes would directly predict actual cheating behavior observed during match play.

**Method:** Adolescent competitive tennis players (*N* = 314, 76.75% males, *M* age = 14.36 years, *SD* = 1.50) completed measures of values, goal orientations, and moral attitudes. A sub-sample (*n* = 90) was observed in 45 competitive tennis matches by trained observers who recorded their cheating and gamesmanship behaviors on a validated checklist.

**Results:** Consistent with hypotheses, athletes’ values predicted their moral attitudes through the effects of goal orientations. Anti-social attitudes directly predicted cheating behavior in actual match play providing support for a direct link between moral attitude and actual behavior.

**Conclusion:** The present study findings support key propositions of Lee and colleagues’ model, and extended its application to competitive athletes in actual match play.

## Introduction

Whether sports participation builds moral character and fosters moral functioning is a matter of debate (Shields and Bredemeier, 2007). Research has indicated that some athletes engage in behaviors that can be directly or indirectly classified as immoral or ethically inappropriate, such as injuring an opponent, cheating, retaliating to a foul, or faking an injury, or engage in behaviors that will psychologically distract or upset the opponents (Long et al., 2006; Boardley and Kavussanu, 2007; Lee et al., 2007, 2008). Over the last 20 years, research has addressed the social psychological constructs that predict moral functioning in sports and emphasized the roles of values, achievement goals, social attitudes and sportspersonship behaviors across age groups, sport levels, and settings (e.g., Duda et al., 1991; Kavussanu and Roberts, 2001; Kavussanu et al., 2006; Sage et al., 2006). The present study builds on this prior research applying a social psychological theory to explain morally or ethically inappropriate behavior in sport (Lee et al., 2008). Specifically, we applied the theory to explain relations between values, achievement goals, and moral attitudes in a sample of young competitive tennis players. Our research also extended evidence for the model by applying it to explain observed actual cheating behavior in a competitive game situation.

### Moral Behavior: Sportspersonship, Cheating, and Gamesmanship

Questions about moral behavior in sports are essentially concerned with how athletes conduct themselves when engaging in their sport (e.g., whether they respect rules and officials or comply with conventions). Developing an equivocally accepted definition of moral behavior in sports is difficult; definitions, operationalization, and interpretation of moral behavior vary according to the origin and perspective of those involved. One line of research has defined moral behavior in relation to sportspersonship, a concept that describes a range of honorable behaviors, including fair play, respecting the rules, respecting the opponents and officials, and accepting defeat and victory (Vallerand et al., 1997; Siedentop et al., 2004). Athletes with high moral functioning are expected to display high scores on indices of sportspersonship, whereas athletes with low moral functioning will score low by comparison and are expected to be more prone to morally questionable behaviors, such as displaying aggression, breaking the rules, and cheating (Ommundsen et al., 2003). Vallerand et al. (1997) developed a social psychological approach to the study of sportspersonship, and argued that moral behavior is multidimensional and should be understood both in terms of individual and contextual characteristics. The basic constructs from their model were assessed on the multidimensional sportspersonship orientation scale (MSOS), which comprises five dimensions: (1) commitment to sports participation, (2) respect for social conventions, (3) rules, (4) officials and opponents, and (5) acceptance of winning at all costs.

Extending the work of Vallerand et al. (1997) and Lee et al. (2007) developed the Attitudes towards Moral Decision Making in Sport Questionnaire (AMDYSQ). This questionnaire tapped some of the key facets of MSOS but also distinguished between antisocial (e.g., acceptance of cheating and gamesmanship) and prosocial attitudes (i.e., keeping winning in proportion). Cheating and gamesmanship was conceptualized as two distinct behaviors that relate to the study of moral behavior in sports. Both behaviors are goal-directed and aim to yield illegitimate benefits. Whereas cheating features explicit rule-violation acts (e.g., doping), gamesmanship represents more subtle morally questionable behaviors that are at odds with sports ethics and aim to psychologically upset the opponent, without de jure violation of the rules of the game (e.g., upset opponents by mocking their errors) (Lee et al., 2007; Ntoumanis and Standage, 2009; Ponseti et al., 2012).

### Attitudes as Value-expressive Constructs

Over the years, Lee et al. (2000, 2007, 2008) proposed that values guide decision-making and behaviors in sport across situations, and that values regarding achievement and morality are prominent factors impacting moral behavior in youth sport. Consistent with this orientation and with classical theorizing on value systems (e.g., Webb, 1969; Schwartz, 1992), Lee et al. (2000, 2007, 2008) classified sport values in adolescent athletes, distinguishing between moral (e.g., obedience, fairness, sportspersonship), competence (e.g., mastery of skills), and status values (e.g., public image, winning, and outdoing others). Moral, competence and status values can influence athletes’ attitudes (evaluative beliefs and/or outcome expectancies relevant to a specific behavior) and choices with respect to different behavioral alternatives. Furthermore, Lee et al. (2007, 2008) explicitly distinguished between values and attitudes and viewed attitudes as bipolar evaluative beliefs that are specific to an attitude object, as well as value-expressive constructs (i.e., attitudes may reflect the expression of certain values). In support of this hypothetical framework, Lee (1996) found that moral values positively predicted prosocial attitudes (i.e., commitment to participation and respect for social conventions) and negatively predicted antisocial attitudes (i.e., acceptance of cheating and gamesmanship) in sport contexts. Likewise, Lee et al. (2008) found that moral and competence values predicted prosocial attitudes positively, whereas moral values were negatively associated with antisocial attitudes. They also found that status values positively predicted antisocial attitudes.

### From Values to Achievement Goals

Lee et al. (2008) value-expressive model of moral attitudes further proposed that competence, moral, and status values influence young athletes’ prosocial and antisocial attitudes via the mediating effects of athletes’ goal orientations. Broadly speaking, goal orientations in sport are concerned with the ways athletes conceive success in their sport activities. According to Achievement Goal Theory (AGT) (Nicholls, 1989), the pursuit of achievement is related to the display of competence and to personal perceptions of success. Athletes may use either self-referenced or other-referenced criteria to personally evaluate their competence and success in sport. Those using self-referenced criteria are referred to as task-oriented and generally interpret success through mastery of tasks and self-improvement, whereas athletes utilizing other-referenced criteria are termed ego-oriented and tend to define success as a function of winning and outperforming others through social comparative processes (Kavussanu and Ntoumanis, 2003; Chatzisarantis et al., 2016).

These two types of goal orientations have been shown to be conceptually and empirically related to the value systems that underpin moral behavior in sport, and prosocial and antisocial attitudes. With respect to values, existing research has shown that task-oriented individuals assign relative importance to, and are predominantly regulated by competence values, whereas, ego-oriented individuals are driven by and regulate their behavior based on status values (Nicholls, 1989; Biddle et al., 2003). Accordingly, existing research has shown that task-oriented athletes tend to display higher sportspersonship behaviors than ego-oriented athletes, and ego-oriented athletes are more likely to endorse morally transgressive behaviors, such as the approval of intentional injurious acts (e.g., Duda et al., 1991; Boardley and Kavussanu, 2010). Similarly, Kavussanu and Ntoumanis (2003) found that task-oriented athletes were more likely to display higher levels of moral functioning and behaviors, and Gonçalves et al. (2010) showed that task orientation was positively related to respect for conventions and commitment, whereas ego orientation was positively related to cheating and gamesmanship, and inversely related to respect for social conventions.

Taken together, the aforementioned evidence is in line with Lee et al.’s (2008) value-expressive model of moral attitudes in youth sport, in which goal orientations are assumed to mediate the effects of values on young athletes’ prosocial and antisocial attitudes. Specifically, Lee et al. (2008) found that task orientation mediated the effect of competence values on young athletes’ prosocial attitudes, ego orientation mediated the effect of status values on antisocial attitudes, and moral values directly predicted attitudes without any mediation by achievement goals. This social psychological approach provides mechanistic explanations of the process by which values determined moral attitudes. Specifically, it implies that individuals with high competence values tend to orient their motivational orientations with respect to interpreting success due to personal improvement on the task, which leads them to adopt attitudes reflecting personal improvement and excellence in their moral performance in sport contexts. In contrast, individuals with high status values are more likely to endorse motivational orientations in which success is interpreted as winning and outperforming others, which means they are more likely to adopt attitudes consistent with a “winning at all costs” perspective, including positive attitudes toward antisocial behaviors in sport context including cheating and gamesmanship.

### The Present Study

The present study intended to apply Lee et al. (2008) value-expressive model of moral attitudes to youth sport. The research makes a unique contribution to knowledge on the social psychological antecedents of moral behavior in sport by examining the predictive validity of the model using externally validated observational measures of cheating and gamesmanship. The research is also the first to apply Lee et al.’s model in a different sport context, namely, highly competitive national youth tennis. We pursued two empirical objectives. First, we aimed to replicate the model in a large representative sample of young competitive Italian tennis players. Specifically, we hypothesized that moral, competence and status values would predict prosocial and antisocial attitudes, and that this effect would be mediated by goal orientations. The rationale for this objective is based on Lee et al.’s (2008) recommendation to apply their model to different populations. Second, we aimed to assess whether moral attitudes predicted actual cheating and gamesmanship behavior within the framework of the Lee et al.’s (2008) model in a sub-sample of competitive tennis players. This is an important addition to the extant research on moral behavior in sports; our study is the first that addresses the issue of attitude–behavior consistency in the context of actual cheating behavior in sports. For this purpose, independent observers recorded instances of cheating and gamesmanship during actual tennis matches. It was hypothesized that prosocial attitudes would be negatively related to displays of cheating and gamesmanship behaviors, whereas antisocial attitudes would be positively related to the likelihood of presenting cheating and gamesmanship behaviors.

## Materials and Methods

### Participants and Procedures

Adolescent tennis players (*N* = 314; 76.75% male, *M* age = 14.36 years, *SD* = 1.50, range: 12–17 years) agreed to participate in the study. Data were collected from mid-December 2011 to the beginning of January 2012. All players were recruited from the LEMONBOWL in Rome, one of the most important international tennis tournaments for young competitive tennis players in the world, with an annual participation of approximately 2000 players. Participants were randomly selected from the roster of Italian adolescent players (nearly 920 athletes). All the athletes who were approached agreed to participate to the study. Consent from the athletes’ parents and the athletes’ themselves was obtained prior to data collection. The study was approved by the Ethics Review Board of the Department of Social and Developmental Psychology, “La Sapienza” University of Rome, and participants were informed of the aims and purpose of the study, as well as their participation rights (e.g., confidentiality of responses, allowance to leave the study at any point without any consequences), in advance of data collection. All participating athletes individually completed study measures in a single questionnaire booklet administered in a quiet room located near the tennis courts. Tennis players were instructed that they could use as much time as needed to complete the survey. Participants typically spent approximately 25 min completing the survey. During the administration sessions, the participants were also informed that their matches during the tournament might have been randomly selected for an additional observation phase of the study.

### Measures

The survey comprised a battery of self-report psychometrically sound measures. The original English versions of the measures were translated into Italian using accepted back-translation techniques (Hambleton, 2001).

#### Values

Values were assessed with the translated “Youth Sport Values Questionnaire” (YSQV-2; Lee et al., 2008). The questionnaire comprised the common stem: ‘*What is important to me in sports…*’ followed by thirteen items measuring moral (5 items, e.g., “Showing good sportsmanship”), competence (4 items, e.g., “Improving my performance”), and status (4 items, e.g., “Being better than others”) values. Responses were recorded on the original seven-point scales^1^, ranging from extremely important to me (5), to the opposite of what I believe (-1). Higher scores reflected stronger values. Previous studies (Lee et al., 2008) supported the threefold factorial structure of the YSQV-2, and found that its subscales exhibited satisfactory internal consistency (Cronbach alphas = 0.79, 0.74, and 0.82 for the moral, competence, and status values scales, respectively) and test–retest reliability (correlations over 4 weeks ranged from 0.66 to 0.72).

#### Achievement Goals

We assessed achievement goals using the Italian version of Task and Ego Orientation in Sport Questionnaire (Bortoli and Robazza, 2005). The questionnaire comprised the common stem: ‘*I feel most successful in sport when …*’ followed by 13 items measuring task orientation (7 items, e.g., “I learn a new skill by trying hard”) and ego orientation (6 items, e.g., “I am the only one who can do the task or skill”). Responses were recorded on five-point Likert scales ranging from strongly disagree (1) to strongly agree (5). Higher scores on each scale reflected greater achievement goals. The Italian version of the TEOSQ was developed by Bortoli and Robazza (2005), who confirmed the original two-factor structure and adequate reliability of the task (Cronbach’s alpha = 0.73) and ego (Cronbach’s alpha = 0.85) orientation subscales.

#### Prosocial Moral Attitudes

Prosocial attitudes were assessed with a translated version of the two MSOS subscales: Commitment to Sport Participation (5 items, e.g., “I go to every practice session”) and “Respect for Social Convention” (5 items, e.g., “I shake hands with the opposition—win or lose”) (Vallerand et al., 1997). Responses were recorded on five-point scales ranging from strongly disagree (1) to strongly agree (5) with higher scores on both scales reflecting stronger prosocial attitudes. The factorial validity of the MSOS was originally evaluated by Vallerand et al. (1997). They also reported adequate internal consistency of the subscales (Cronbach’s alpha = 0.71, and 0.86, respectively, for the Commitment in Sport Participation and Respect of Social Convention subscales). Test–retest reliability coefficients over 5 weeks were 0.76 for both subscales (Vallerand et al., 1997).

#### Antisocial Moral Attitudes

Antisocial attitudes were assessed using the Italian version of the Attitudes to Moral Decision-Making in Youth Sport Questionnaire (AMDYSQ; Lee et al., 2007), including the Acceptance of Cheating” scale (4 items, e.g., It is OK to cheat if nobody knows) and the “Acceptance of Gamesmanship” scale (5 items, e.g., “I sometimes try to wind up the opposition”). All responses were recorded on five-point scales, ranging from strongly disagree (1) to strongly agree (5). Higher mean scores for both subscales reflected stronger antisocial attitudes. Lee et al. (2007) reported support for the factorial validity and internal consistency (Cronbach alpha = 0.73 and 0.75, respectively, for the Acceptance of Cheating and Acceptance of Gamesmanship subscales, respectively) of the measure.

#### Cheating and Gamesmanship Behavior in the Field

We used an observation protocol to record cheating and gamesmanship behaviors during competitive tennis matches in a subsample (*n* = 90) of young tennis players randomly selected from the total sample. The choice of selecting a subsample of young tennis players’ matches was determined by the number of trained observers available (*n* = 8) at any one time to observe and code tournament matches. In fact, the maximum number of matches that could be subjected to observation was 45. It should be noted that, as in other youth tennis tournaments, the LEMONBOWL matches do not have an official umpire and players are required to officiate their own matches. **Table 1** shows the list of cheating and gamesmanship behaviors assessed in the study and the number of tennis players who displayed each behavior at least once during the matches. The behaviors listed in **Table 1** were selected based on data from a focus group interview. The focus group included six experienced and Masters-level tennis players who were asked to generate and reach consensus about instances of cheating and gamesmanship behaviors that typically occur during tennis matches in youth tennis tournaments. Their data was used to generate cheating and gamesmanship items for the observation protocol. Subsequently, an additional and independent group of six tennis experts was involved to verify the face and content validity of the items in the observation protocol. Specifically, the experts were initially provided with the operational definition of cheating and gamesmanship, and then asked to evaluate the soundness and representativeness of the instances of cheating and gamesmanship included in the observation protocol. Each expert in this panel individually confirmed the soundness and representativeness of the behaviors listed in **Table 1**.

**Table 1:** Number of players displaying specific cheating and gamesmanship behaviors observed during a competitive match.

Observed behaviors	*n*
Cheating (at least one of the following cheating behaviors)	19
(1) Call “out” a good or uncertain ball of the opponent.	19
(2) Call a score different from the real one.	10
(3) Call an uncertain “net” on the opponent’s service to its own advantage.	1
(4) Do not report a own field invasion or a own touch of the ball with their body.	2
(5) Delete a sign of a doubtful ball.	1
(6) Indicate on the ground a different sign of the ball.	6
Gamesmanship (at least one of the following gamesmanship behaviors)	21
(1) Rejoice for a mistake of the opponent.	18
(2) Lose time during field changes (the opponent is ready and waits).	7
(3) Stop the opponent during his service (raising the racquet, tying his shoes, etc.).	6
(4) Stop the game for several reasons (bathroom, illness, change of racquet, etc.).	1
(5) Denigrate the opponent explicitly or implicitly during the game.	6
(6) Call aloud the score only when it is in his favor.	3
(7) Argue with the opponent during the change of the field (when game is stop).	1
(8) Resend violently on the opponent’s field a service clearly “out”.	18

*The cheating and gamesmanship behavior types were not mutually exclusive, so players could display more than one type of behavior during the match.*

The eight observers, all tennis experts, were trained on how to recognize and record cheating and gamesmanship using the observation protocol and record them on the checklist (see **Table 1**). Observers were initially provided with the operational definition of each behavior, and were presented with a demonstration of each behavior on a tennis court. They were then trained on how to use the checklist during trial tennis games that simulated the LEMONBOWL tournament. For the actual behavioral assessment, 45 tennis matches from the tournament were randomly selected, and the two players involved in each of these matches were observed by a pair of observers, randomly assigned to observe the match. Each observer individually recorded, in real time, (a) the occurrence of cheating or gamesmanship behaviors displayed by the two players and, for each recorded behavior, and (b) the exact moment and match score when the behavior occurred on the checklist (these latter data were not used in the present study). For each behavior listed on the observation tool, observers assigned the player a “yes” score if they enacted the target behavior at least once during the match, or “no” if they did not enact that behavior during the match. Therefore, players receiving a “yes” one or more times across the listed cheating behaviors were classified overall as 1 = “engaged in cheating behaviors”, while players that did not register a “yes” for any of the listed cheating behaviors were scored as 0 = “did not engage in cheating behaviors”. The same procedure was applied to gamesmanship behaviors. Inter-rater reliability was calculated across all matches and showed a relatively high level (Cohen’s Kappa for each pair of observers ranged between 0.85 and 1.00). The limited cases of discrepancy in the two observers’ ratings were resolved by discussing the cases after the matches and by reaching a consensus. The use of observational checklists to measure behavior is a common approach in social science research (e.g., Cale, 1994; McLachlan and Hagger, 2010).

### Data Analysis

Data were analyzed using structural equation modeling (SEM) with the Mplus statistical software (Muthén and Muthén, 2010). In our first analysis, we tested a “replication” model in which we aimed to replicate the key findings of Lee et al. (2008) model of prosocial and antisocial attitudes in youth sport. In a second analysis, we tested a “behavioral” model in which we extended Lee et al. (2008) model to the prediction of actual moral behavior in sports contexts. Specifically, our model specified that young tennis players’ prosocial and antisocial attitudes would predict cheating and gamesmanship behaviors based on our observational measure during the actual matches. Model parameters were estimated using the Maximum Likelihood (ML) estimation method for the “replication” model (Model 1), whereas the Weighted Least Squares Means and Variance adjusted (WLSMV) estimation methods, which are suitable for data with categorical variables (Muthén and Muthén, 2010) (Cheating: Yes or no; Gamesmanship: Yes or no), were used for the extended “behavioral” model (Model 2). Furthermore, we parceled items from the questionnaires together to produce indicators of the latent factors of the study constructs using recommended procedures (Kim and Hagtevt, 2003). This was to reduce the dimensionality and the number of parameters of the models, resulting in more parsimonious and more stable measurement estimates (Little et al., 2002). More specifically, in the present study, parcels for each latent variable were created by randomly grouping the items of each scale into three separate item sets (parcels) and by averaging the item scores within each set.

**FIGURE 1:**
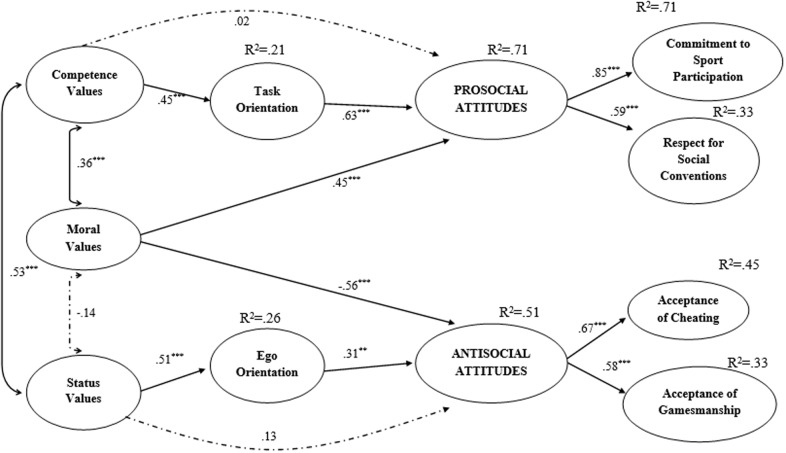
**Structural equation model of hypothesized relations in the ‘replication model’ on data from the first phase of the study (*N* = 314).** Values are standardized coefficients. ^∗^*p* < 0.05, ^∗∗^*p* < 0.01, ^∗∗∗^*p* < 0.001. Dotted lines indicate non-significant parameters

**FIGURE 2:**
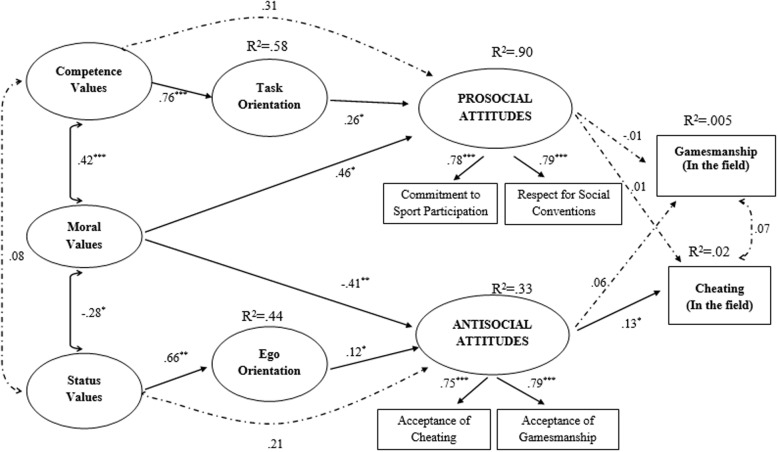
**Structural equation model of hypothesized relations in the ‘behavioral model’ on data from the second phase of the study (*n* = 90).** Values are standardized coefficients. Values are standardized coefficients. ^∗^*p* < 0.05, ^∗∗^*p* < 0.01, ^∗∗∗^*p* < 0.001. Dotted lines indicate non-significant parameters.

Focusing on Model 1, consistent with the Lee et al.’s (2008) hypothesized model, we proposed that athletes’ competence, moral, and status values would be related with both social (i.e., commitment to sport participation and respect for social convention) and antisocial (i.e., cheating and gamesmanship) attitudes, and that athletes’ task and ego orientations would, respectively, be related with prosocial and antisocial attitudes. We also hypothesized that athletes’ task orientation would mediate the relationship between competence values and prosocial attitudes, while ego orientation would mediate the relationship between status values and antisocial attitudes, consistent with Lee et al.’s (2008) model. A diagram of the model is presented in **Figure 1**. We followed Preacher and Hayes’ (2008) procedures to evaluate out mediation hypotheses. Specifically, we calculated the indirect effects and their 95% confidence intervals using a bootstrapped resampling method with 5000 resamples. Mediation was confirmed by the presence of a statistically significant bootstrapped indirect effect. Focusing on Model 2, we augmented the direct and indirect effects in Model 1 with four additional paths in which prosocial and antisocial attitudes were proposed to predict cheating and gamesmanship behaviors from participants’ actual tennis matches based on scores on the observational checklist. The diagram for Model 2 is presented in **Figure 2**.

There are two important addenda to the above analyses. First, as **Figures 1**, **2** shows, the measurement models of athletes’ prosocial and antisocial attitudes were different across the two analyses. In Model 1, both types of social attitudes were hypothesized to indicate a second-order factor structure in which indicators were developed using the item parceling procedure. In contrast, in Model 2, we used a first-order factor structure in which indicators of prosocial and antisocial attitudes were identified by aggregating the observed scores utilized in the first analysis. These choices were made in lieu of the reduction in sample sizes across analyses: the replication model was tested in a relatively large sample (*N* = 314) while the behavioral model was tested in a substantially smaller subsample (*n* = 90). Second, scores for cheating and gamesmanship behaviors from the observation checklist were treated as dichotomous. In line with recommendations by Muthén and Muthén (2010), the metrics of the first and second order factors of the model were automatically defined by setting one arbitrary factor loading to unity in order to avoid identification problems.

## Results

Descriptive statistics, internal consistency reliability coefficients, and intercorrelations for the study measures in the whole sample (*N* = 314) are presented in **Table 2**. Intercorrelations for the study measures in the sub-sample (*n* = 90) are reported in **Table 3**.

**Table 2:** Descriptive statistics, reliability coefficients, and intercorrelations for study variables in the whole sample (*N* = 314).

		1	2	3	4	5	6	7	8	9
1	Moral values	–								
2	Competence values	0.31^∗∗^	–							
3	Status values	–0.10	0.29^∗∗^	–						
4	Task orientation	0.39^∗∗^	0.30^∗∗^	0.02	–					
5	Ego orientation	–0.17^∗^	0.05	0.45^∗∗^	0.05	–				
6	Commitment to sport participation	0.41^∗∗^	0.40^∗∗^	–0.01	0.62^∗∗^	–0.04	–			
7	Respect for social convention	0.54^∗∗^	0.22^∗∗^	–0.15^∗^	0.38^∗∗^	–0.17^∗^	0.48^∗∗^	–		
8	Acceptance of cheating	–0.32^∗∗^	0.05	0.26^∗∗^	–0.21^∗∗^	0.24^∗∗^	–0.27^∗∗^	–0.27^∗∗^	–	
9	Acceptance of gamesmanship	–0.28^∗∗^	–0.00	0.17^∗^	–0.18^∗^	0.23^∗∗^	–0.12^∗^	–0.18^∗^	0.50^∗∗^	–
	Mean ±*SD*	3.92 ± 0.70	4.33 ± 0.63	2.25 ± 1.22	4.33 ± 0.51	2.56 ± 0.73	4.35 ± 0.61	4.2 ± 0.69	1.46 ± 0.63	2.21 ± 0.83
	Skewness/Kurtosis	–0.79/1.27	0.62/1.61	1.22/–0.42	–0.1.04/1.44	0.30/–0.22	–1.41/2.43	–1.13/1.01	1.66/2.43	0.48/–0.27
	Alpha based on all items of the scale	0.63	0.66	0.79	0.78	0.78	0.77	0.77	0.76	0.76
	Alpha based on SEM item parcels	0.57	0.60	0.68	0.72	0.76	0.73	0.76	0.74	0.86

*^∗^p < 0.05, ^∗∗^p < 0.01, ^∗∗∗^p < 0.001.*

**Table 3:** Intercorrelations among study variables for the sub-sample (*n* = 90).

		1	2	3	4	5	6	7	8	9	10
1	Moral values	–									
2	Competence values	0.54^∗∗^	–								
3	Status values	–0.19	0.16	–							
4	Task orientation	0.56^∗∗^	0.40^∗∗^	0.06	–						
5	Ego orientation	–0.19	–0.06	0.43^∗∗^	–0.01	–					
6	Commitment to sport participation	0.55^∗∗^	0.55^∗∗^	0.01	0.68^∗∗^	–0.04	–				
7	Respect for social convention	0.60^∗∗^	0.46^∗∗^	–0.16	0.43^∗∗^	–0.15	0.58^∗∗^	–			
8	Acceptance of cheating	–0.44^∗∗^	0.01	0.22^∗^	–0.23^∗^	0.10	–0.26^∗^	–0.37^∗∗^	–		
9	Acceptance of gamesmanship	–0.35^∗∗^	–0.03	0.16	–0.22^∗^	0.35^∗∗^	–0.12	–0.24^∗^	0.60^∗∗^	–	
10	Observed cheating	–0.05	0.07	0.10	–0.03	0.07	–0.10	–0.03	0.09	0.00	
11	Observed gamesmanship	–0.01	0.06	0.10	–0.02	–0.02	–0.07	–0.03	0.02	–.04	0.04
	Mean ±*SD*	3.88 ± 0.80	4.36 ± 0.58	2.46 ± 1.13	4.24 ± 0.58	2.64 ± 0.71	4.24 ± 0.73	4.09 ± 0.81	1.54 ± 0.70	2.24 ± 0.87	–
	Skewness/Kurtosis	–1.26/2.84	–0.79/–0.02	0.01/–0.52	–0.809/0.73	0.11/–00.42	–1.26/1.38	–0.85/0.03	1.65/2.86	0.47/–0.29	–
	Alpha based on all items of the scale	0.74	0.60	0.74	0.82	0.74	0.83	0.80	0.80	0.84	–
	Alpha based on SEM items parcels	0.72	0.59	0.64	0.83	0.76	0.86	0.81	0.83	0.80	–

*^∗^p < 0.05, ^∗∗^p < 0.01, ^∗∗∗^p < 0.001.*

### “Replication” SEM Model

Overall, goodness-of-fit indices suggested indicated that the measurement characteristics of the replication model adequately accounted for the data (Model 1; χ^2^_(299)_ = 472.57, *p* = 0.001; CFI = 0.93; TLI = 0.90; RMSEA = 0.04, 90% CI RMSEA = 0.04 to 0.05; SRMR = 0.06). The factor loadings of the latent constructs were statistically significant and the average variance extracted (AVE) was significantly different from zero for the moral values (0.31), competence values (0.36), status values (0.43), task orientation (0.48), ego orientation (0.51), prosocial attitudes (0.85), and antisocial attitudes (0.67) factors, respectively.

The structural model also demonstrated a good fit with the data (χ^2^_(309)_ = 516.42, *p* = 0.001; CFI = 0.91; TLI = 0.90; RMSEA = 0.05, 90% CI RMSEA = 0.04 to 0.05; SRMR = 0.07). Standardized path coefficients for the hypothesized pathways in the model indicated that moral values were negatively and directly associated with tennis players’ cheating and gamesmanship attitudes (β = –0.56; *p* < 0.001). They were also directly and positively associated with athletes’ prosocial attitudes (β = 0.45; *p* < 0.001). In contrast, competence and status values had no direct associations with prosocial (β = 0.02, *p* = 0.87) and antisocial (β = 0.13, *p* = 0.27) attitudes, whereas they were positively associated with task (β = 0.45; *p* < 0.001) and ego (β = 0.51, *p* < 0.001) orientations, respectively. Task and ego orientations were associated, respectively, with prosocial (β = 0.63, *p* < 0.001) and antisocial attitudes (β = 0.31, *p* < 0.001). Finally, the indirect effect of competence values on prosocial attitudes through task orientation was statistically significant (β = 0.29, *p* < 0.001, 95% CI = 0.16 to 0.44). Similarly, the indirect effect of status values on antisocial attitudes through ego orientation was statistically significant (β = 0.16; *p* < 0.001, 95% CI = 0.03 to 0.29).

### “Behavioral” Model

Turning to the behavioral model, the measurement model showed adequate fit with the data (χ^2^_(131)_ = 170.736, *p* = 0.01; CFI = 0.93; RMSEA = 0.06; SRMR = 0.08). The AVE for the latent variables were statistically significant for the moral values (0.47), competence values (0.24), status values (0.34), task orientation (0.62), ego orientation (0.52), prosocial attitudes (0.58) and antisocial attitudes (0.59), respectively.

The structural model showed an adequate fit statistics (χ^2^_(175)_ = 203.02, *p* = 0.07; CFI = 0.91; RMSEA = 0.042; Weighted Root Mean Square Residual = 0.74). As reported in **Figure 2**, standardized path coefficients indicated an identical pattern of effects among players’ values, goal orientations, and social attitudes to that found in Model 1 with the entire sample, consistent with Lee et al.’s (2008) model. Importantly, the analysis yielded a statistically significant direct effect of tennis players’ antisocial attitudes on observed cheating behaviors (β = 0.13, *p* = 0.019). There were no statistically significant direct effects of prosocial (β = –0.01, *p* = 0.93) and antisocial (β = 0.06, *p* = 0.77) attitudes on observed gamesmanship behaviors, or of prosocial attitudes on cheating behavior (β = 0.01, *p* = 0.96). Finally, with respect to possible indirect effects of sport values on cheating behavior, the indirect effect of moral values on cheating behavior through the antisocial attitudes (β = –0.055, *p* = 0.88, 95% CI = –0.80 to 0.69), and the indirect effect of status values on cheating behavior through ego orientation and prosocial attitudes (β = 0.011, *p* = 0.97, 95% CI = –0.58 to 0.60), were not statistically significant.

## Discussion

The purpose of the present study was to replicate Lee et al.’s (2008) model of values-goal orientations-moral attitudes in a sample of young Italian tennis players, and examine the effects of moral attitudes on actual cheating and gamesmanship behaviors, observed during actual tennis matches.

With respect to our aim to replicate Lee et al.’s model, findings demonstrated that competence and status values indirectly influenced young athletes’ prosocial and antisocial attitudes through the mediating effects of their task and ego orientations. Furthermore, moral values also directly influenced athletes’ prosocial and antisocial attitudes. These findings were consistent with Lee and colleagues’ model, and extended its application to competitive young tennis athletes, thus strengthening the model’s tenability and generalizability in a different cultural context (i.e., Italy) and sport contexts (i.e., individual sport, such as tennis).

With respect to our aim of extending the model findings to objective measures of cheating behavior, findings indicated that moral attitudes exerted a direct, albeit small, effect on young tennis athletes’ cheating behaviors during actual tennis matches. This finding is also consistent with Lee et al.’s (2008) model, and extends the predictive validity of the model in sport contexts by corroborating the general notion that moral behavior (i.e., cheating behaviors in tennis competitive matches) is related to social cognitive determinants, such as moral attitudes. To the best of our knowledge, no study to date has evaluated the links between moral attitudes and cheating or gamesmanship behaviors in sport.

Taken together, the findings of the present study are important for three sets of reasons. First, although it provides a well-known theoretical basis for linking values, attitudes and behavior, the value-expressive model of attitudes (Lee et al., 2008) had not been tested on actual cheating or gamesmanship behavior in sport. The present research provided the first instance of such a test.

Second, our findings imply that, at least to some degree, the display of morally questionable behaviors (i.e., cheating) is guided by athletes’ antisocial attitudes about moral behavior and conduct in sport settings. This notwithstanding, the lack of any effects from prosocial attitudes to behavior does not rule out the possibility that the attitude-behavior relation, especially concerning gamesmanship behaviors, may also depend on social psychological antecedents that were not measured in the present study. In this regard, it also seems relevant to point out that, while cheating behaviors typically draw upon, and are evaluated with respect to, clear, explicit and well-defined sets of rules, gamesmanship behaviors call upon an individual’s personal representations of sport rules and codes of conduct. This distinction may well have had some impact on the differences in findings across cheating and gamesmanship behaviors.

Third, given that cheating behavior is generally viewed as socially and culturally undesirable (Lee et al., 2007; Ponseti et al., 2012), self-reports measures of cheating and gamesmanship behaviors may be particularly sensitive to social desirability and impression management biases. Part of the present research relied on an observational behavioral measure that provided an externally validated, objective assessment of cheating, and gamesmanship in actual competitive matches that was not subject to social desirability and reporting bias. This methodological choice circumvented the heavy-reliance on self-reported data of cheating and gamesmanship behaviors that tends to characterize the scientific literature on moral behavior in sport (e.g., Duda et al., 1991; Kavussanu and Roberts, 2001; Kavussanu et al., 2006; Sage et al., 2006; Boardley and Kavussanu, 2010). The findings partly supported this choice and provided, for the first time, evidence as to the linkages between moral attitudes and actual cheating.

The current study may serve as a platform for future research on the theoretical links between prosocial and antisocial attitudes, motivational factors, and cheating and gamesmanship behaviors. For instance, although our data does not allow a direct test of this assumption, findings imply that cheating attitudes guide cheating behavior directly without the need for conscious intention-formation (i.e., the athletes do not need to have formed conscious intentions to cheat before the game). This hypothesis may partly account for the single effect found between antisocial attitudes and cheating (e.g., antisocial attitudes are conceptually quite close and specific to cheating) and, at the same time, for the lack of an attitude-behavior relation in the case of prosocial attitudes. Finally, the lack of any effect of antisocial attitudes on gamesmanship could be related to different levels of perceived acceptability of the two antisocial-behaviors by athletes (i.e., cheating could be perceived as more “antisocial” than gamesmanship). In line with this argument, a mounting body of evidence has shown that features of the context can trigger mental representations, such as attitudes or values, outside of awareness and accordingly drive behavior in an offhand manner (Bargh et al., 2001; Aarts and Dijksterhuis, 2003). This may be a promising approach for future studies on moral behavior in sports, especially among adolescent and young athletes who tend to be more susceptible to contextual and normative influences (Simons-Morton et al., 2009).

In addition, we propose three additional explanations for the non-significant effect of pro-social attitudes on actual gamesmanship and cheating behavior. Firstly, attitude specificity may determine the influence of attitudes on behavior (Ajzen, 1991). More specifically, pro-social attitudes can be seen as distinct from, or orthogonal to, antisocial attitudes. In this respect, pro-social attitudes may be more relevant to the prediction of concomitant moral behaviors (e.g., respect to officials, rules, and conventions, display of fair play in the field), but not predict displays of cheating. Anti-social attitudes may be more related to cheating, than fair play behavior. This argument is in line with the view of pro-social and antisocial attitudes as functionally independent, and not as the two opposing ends of a continuum of moral functioning (see also Cacioppo et al., 1997). A second explanation relates to attitudinal ambivalence (Armitage and Conner, 2000). Athletes may view respect for rules and fair play as socially desirable and beneficial behaviors without any long-term costs, but at the same time maintain that occasionally violating the rules or engaging gamesmanship may yield short-term benefits that could not be derived by fairplay. A related explanation has to do with attitude accessibility and stability. Attitudes are more likely to predict behavior when they are easily accessible and stable over time (Glasman and Albarracin, 2006). Therefore, athletes who displayed cheating behavior may also hold salient, easily accessible, and stable positive attitudes towards cheating. Although our data do not allow for a direct test of this assumption, the issue of attitude salience and accessibility is worth exploring in future research on moral attitudes in youth sports.

In terms of theoretical implications, our replication of Lee et al.’s (2008) findings indicated that the process by which values are related to pro- and antisocial attitudes in sport occurs via the adoption of achievement goals (Gonçalves et al., 2010). Specifically, the association of competence values with the adoption of prosocial attitudes is mediated by athletes’ views that success in sport is concerned with the mastery of sport tasks. In contrast, status values may elicit antisocial attitudes via the intervening effects of athletes’ views that success in sport is concerned with outperforming and comparing oneself to others. These possibilities provide a motivational basis for the development of pro- and anti-social attitudes in sport. A general hypothesis derived from these findings might be that values influence motivational orientations about success in sport which, in turn, affect athletes’ attitudes about moral behaviors. In other words, one of the reasons why athletes with particular values have particular attitudes about what is right or ‘acceptable’ and what is ‘wrong’ or unacceptable in sport comes down to the ways they interpret success in sport (Kavussanu and Ntoumanis, 2003; Chatzisarantis et al., 2016). Thus, if one values status and focuses on “success as performance”, he or she may endorse a ‘win at all costs’ attitude and acquire a relatively stronger acceptability of antisocial or immoral behaviors, like cheating. In contrast, if an athlete values competence and focuses on “success as mastery of specific sport tasks”, he or she may hold an attitude for which ‘playing the game well’ is a relevant indication of personal competence and this attitude may well imply accepting and conforming to rules and morally appropriate codes of conduct.

Another important implication is concerned with the generalizability of the present research’s findings. Our findings extend those Lee et al. (2008) to young high-level competitive Italian tennis players. This generalizability seems to occur even though considerable cultural differences exist between that in which the model was original developed and the Italian culture. However, this should not be a surprise as even though moral codes my vary across national group, the context in which athletes train and compete is separate and may share many characteristics with similar contexts in other nations. Thus, the current findings may point to the fact that young athletes that spend a lot of time in sport contexts may hold more homogenous values than similar aged non-athletic young people who are not exposed to the values in sport contexts and, instead, are more likely express the general values endorsed by general society.

### Limitations

The present study was cross-sectional in design, which means we cannot infer causal relations among the constructs from the data, only from theory. Longitudinal designs, particularly cross-lagged panel designs may assist researchers in addressing hypotheses of the value-expressive model of moral attitudes in youth sport. In addition, although we systematically developed our observational checklist for cheating and moral behaviors in sport, we did not conduct a large-scale validity trial. It would be important that future research to corroborate our small-scale development of the measure on a larger sample with more raters. This limitation notwithstanding, we attained good inter-rater reliability and expert corroboration of face validity of our measure. Finally, the measures used to tap the model value constructs in the current study from the MSOS and the AMDYS, did not exhibit robust solution estimates (e.g., AVE < 0.50). It may be the meaning of the constructs in contexts and cultural settings other than those in which the scales were originally developed may vary. Current results should be interpreted in light of the suboptimal validity of these scales and revision and confirmation of the construct validity of these scales in multiple contexts and cultures is warranted. In addition, we were unable to replicate Lee et al.’s (2008) test for gender differences in model relations due to the low number of female athletes in the present sample. Finally, other characteristics such as athletes’ level of sport involvement (e.g., professionals vs. amateur) or contextual factors (e.g., the presence of a referee or not on the tennis court) might have moderated the effects in the model. Such moderating effects were not addressed in the present study and we look to future research to examine these behavioral effects in other sports (e.g., football, basketball).

### Implications for Educational Programs and Interventions

Despite its correlational nature, the present study can provide some initial considerations as to the ways social agents in youth sport (e.g., coaches, parents) may promote moral attitudes and behavior in young athletes. Lee et al. (2008) suggest that coaches, parents and other relevant stakeholders interested in promoting moral behavior in youth sports should focus on and foster moral and competence values, rather than status values. They advocate that moral and competence values foster prosocial attitudes in sport through a view of achievement and success that is based on personal standards, effort and mastery (i.e., goal task orientation). Data from the present study corroborates this call and demonstrates their relevance to the design of educational programs and interventions at the competitive level. Furthermore, coaches and parents may also consider fostering achievement goal orientations, which may lead to young athletes developing adaptive mastery orientations, personal standards and effort-based success in sport.

## Conclusion

The present research provided empirical support for Lee et al. (2008) model in a large sample of Italian young tennis players and extended them to actual cheating and gamesmanship behaviors corroborated by observation. The research also demonstrates the mechanisms by which attitudes affect values lead to pro- and anti-social attitudes through the goals that young people adapt in sport. Our findings pave the way for future longitudinal research on the social psychological antecedents of cheating and gamesmanship behavior in competitive sports and has implications for developing interventions and educational programs to promote moral norms by sports leaders to engender better moral functioning in competitive athletes (Hardcastle et al., 2015).

## Author Contributions

All authors listed, have made substantial, direct and intellectual contribution to the work, and approved it for publication.

## Conflict of Interest Statement

The authors declare that the research was conducted in the absence of any commercial or financial relationships that could be construed as a potential conflict of interest.

## Footnotes

1 Extremely important to me (5), very important to me (4), important to me (3), quite important to me (2), slightly important to me (1), not important to me (0), and the opposite of what I believe (-1).
